# Building an economy for health and well-being 

**DOI:** 10.2471/BLT.24.291719

**Published:** 2024-05-01

**Authors:** Petteri Orpo, Tedros Adhanom Ghebreyesus

**Affiliations:** a Government of Finland, Helsinki, Finland.; b World Health Organization, Avenue Appia 20, 1211 Geneva 27, Switzerland.

The coronavirus disease 2019 (COVID-19) pandemic laid bare the vulnerabilities and inequities engrained in economic and social systems in many countries. This pandemic exposed significant inequalities in access to life-saving vaccines and other medical countermeasures because of insufficient and geographically limited production capacity, economic imbalances and export restrictions, among other factors.^1^ High-risk populations in lower-income countries – including older people, health workers and people with comorbidities – received vaccines long after lower-risk populations in higher-income countries.

In this context, the World Health Organization (WHO) set up the WHO Council on the Economics of Health for All, comprised of 10 leading economists, public health, finance and development professionals. Supported by the Government of Finland, the council issued its final report^2^ in May 2023. This landmark report challenged existing perceptions, debunked old myths, and laid out a roadmap for a world where the health and well-being of people and the planet takes precedence. The report reimagines measures of economic development; highlights the need for improving both the quality and quantity of financing for health and well-being, and equity-focused governance for new vaccines and treatments; and stresses building the necessary capacities in government and society to deliver health for all.

This thematic issue of the *Bulletin of the World Health Organization* is inspired by the council’s work.^3^ This collection of articles is an important contribution to informing creative, collaborative and courageous actions, to reorient economies to value health as an investment in a better future for all. The council argued, citing World Bank and WHO estimates, that the cost of inaction is higher than the cost of action: it would cost 1.30 United States dollars per person on the planet to build an effective global system of pandemic prevention and response that could avoid repeating the experience of COVID-19, whereas the costs of the pandemic were many times higher, including a contraction of global gross domestic product (GDP) by 3.1% between 2019 and 2020. We need to see health not as a cost, but a key driver of sustainable growth.

These actions require a whole-of-government approach to create the conditions in which health can thrive, by addressing the determinants of health and the interlinked challenges we face today. The government of Finland has a long history of cross-sectoral collaboration, and a whole-of-government approach is part of everyday practice. One example is the cross-administrative programme Get Finland Moving,^4^ to increase physical activity and functional ability, improve well-being and reduce the costs incurred by society of sedentary lifestyles. The government implements the programme’s action plan in collaboration with businesses, labour market organizations, civil society and the media.

We recognize that the world needs better mechanisms to facilitate dialogue between ministries of finance, economy and health. Sharing know-how and benchmarking good practices nationally and internationally are important. For this to happen we need structures and operating models that support cooperation, such as health in all policies.^5^ An example is WHO’s accelerated efforts to address the commercial determinants of health, in the marketing of products such as tobacco, ultra-processed foods, fossil fuel and alcohol, which are estimated to account for at least one third of global deaths.^6^

This reorientation also requires radical action to restore the delicate relationship between planet, people, prosperity and equity, based on the recognition that a sustainable economy can be a solid foundation for health and well-being, and in turn, that health and well-being are central factors for sustainable economic growth, social stability and resilience. For Finland, sustainable well-being is based on education and culture, knowledge and competence, respect for work and entrepreneurship, social protection, and non-discrimination and gender equality. 

Investments must protect the health and well-being of young people and future generations, and foster healthy ageing. This goal requires empowering and enabling individuals, families and communities to make healthy choices, where governments put in place participatory processes and ensure credible health information reaches all communities, especially marginalized and vulnerable populations.

Long-term investments in health and well-being can make an important contribution to sustainable economies. An alignment across economic, ecological and social dimensions enables sustainable development, and forms solid and sustainable ways to respond to interlinked crises.

In a world driven by overlapping crises including conflict, climate change and searing inequality, the status quo will not do. We need new models to help us reimagine the future. The work of the council, and of researchers in this issue, helps us to do that and can inform active dialogue between the finance, economic and health sectors.
